# Revisiting Porcine Circovirus Disease Diagnostic Criteria in the Current *Porcine Circovirus 2* Epidemiological Context

**DOI:** 10.3390/vetsci9030110

**Published:** 2022-03-02

**Authors:** Joaquim Segalés, Marina Sibila

**Affiliations:** 1 Unitat Mixta d’Investigació IRTA-UAB en Sanitat Animal, Centre de Recerca en Sanitat Animal (CReSA), Campus de la Universitat Autònoma de Barcelona (UAB), Bellaterra, 08193 Barcelona, Catalonia, Spain; 2 Departament de Sanitat i Anatomia Animals, Facultat de Veterinària, Campus de la Universitat Autònoma de Barcelona (UAB), Bellaterra, 08193 Barcelona, Catalonia, Spain; 3 OIE Collaborating Centre for the Research and Control of Emerging and Re-emerging Swine Diseases in Europe (IRTA-CReSA), Bellaterra, 08193 Barcelona, Catalonia, Spain; marina.sibila@irta.cat; 4 IRTA Programa de Sanitat Animal, Centre de Recerca en Sanitat Animal (CReSA), Campus de la Universitat Autònoma de Barcelona (UAB), Bellaterra, 08193 Barcelona, Catalonia, Spain

**Keywords:** *Porcine circovirus 2* (PCV-2), porcine circovirus disease, clinical signs, pathology, diagnosis, epidemiology, vaccination

## Abstract

Current knowledge on porcine circovirus diseases (PCVD) caused by *Porcine circovirus 2* (PCV-2) includes the subclinical infection (PCV-2-SI), systemic (PCV-2-SD) and reproductive (PCV-2-RD) diseases, and porcine dermatitis and nephropathy syndrome (PDNS). Criteria to establish the diagnosis of these conditions have not changed over the years; thus, the triad composed by clinical signs, lesions and viral detection in lesions are still the hallmark for PCV-2-SD and PCV-2-RD. In contrast, PCV-2-SI diagnosis is not usually performed since this condition is perceived to be controlled by default through vaccination. PDNS is diagnosed by gross and histopathological findings, and PCV-2 detection is not recognized as a diagnostic criterion. Molecular biology methods as a proxy for PCVD diagnoses have been extensively used in the last decade, although these techniques should be mainly considered as monitoring tools rather than diagnostic ones. What has changed over the years is the epidemiological picture of PCV-2 through the massive use of vaccination, which allowed the decrease in infectious pressure paralleled with a decrease in overall herd immunity. Consequently, the need for establishing the diagnosis of PCVD has increased lately, especially in cases with a PCV-2-SD-like condition despite vaccination. Therefore, the objective of the present review is to update the current knowledge on diagnostic criteria for PCVDs and to contextualize the interest of using molecular biology methods in the overall picture of these diseases within variable epidemiological scenarios of PCV-2 infection.

## 1. Introduction

Ten years ago, a review paper on clinical signs, pathology and laboratory diagnosis in relation to *Porcine circovirus 2* (PCV-2) was published, trying to unify existing criteria to establish a proper herd diagnosis of its infection outcomes [1]. At that time, just few years after starting mass vaccination against PCV-2 all over the world [2], it was discovered that PCV-2-sublinical infection (PCV-2-SI) was not only the most frequent form of this viral infection, but also the costliest one [3]. Since then, more than 10 years of a successful story behind PCV-2 vaccination has been contemplated by the swine industry [4,5], to the point that it is difficult to think of producing pigs without vaccination against this pathogen.

Besides the excellent results given by PCV-2 vaccination of piglets [6,7,8], the fact of immunizing this age group of animals has implied to change the epidemiology of this viral infection. The systematic vaccination at weaning implied to significantly decrease the overall herd infectious pressure, and some pig batches may reach almost seronegative at slaughter age or with a low number of animals seroconverting [9]. Therefore, in face of the existing epidemiological changes of PCV-2 infections, it is important to adapt the diagnostic needs to the new scenarios.

Considering that several conditions can cause wasting, respiratory or digestive signs and poor production in pigs, the need for diagnosing porcine circovirus diseases (PCVDs) and to assess their impact on the herd profitability is probably as or even more important than 10 years ago. Hence, the objective of the present review article is to update the criteria used to diagnose infections by PCV-2 and to discuss diagnostic clues within the different epidemiological scenarios in a massively vaccinated swine industry.

## 2. PCV-2 Infection Outcomes and Their Lesions

Gross and microscopic lesions associated with PCVDs are described elsewhere [1]. In fact, the pathological outcome of these diseases has not changed since their initial descriptions [10,11,12,13,14]. It is important to emphasize that the major difference between subclinical versus clinical infections is the severity in the degree of lesions. While subclinical PCV-2 infections are usually correlated with no gross findings and no or mild microscopic lesions, clinical infections (PCV-2-systemic disease, PCV-2-SD, and PCV-2-reproductive disease, PCV-2-RD) are characterized by moderate to severe lesions in the affected tissues (lymphoid tissues in postweaning pigs and heart in the fetus). In addition, the amount of virus load in serum and tissues is usually associated with the lesion severity; the more severe the lesions, the higher the amount of PCV-2 [15]. Recently, a report indicated high viral loads and reproductive problems without myocarditis, the hallmark PCV-2-RD lesion in fetuses [16]. However, other studies have consistently detected such lesions linked to a high amount of PCV-2 in heart tissue [17,18,19].

## 3. PCV-2 Infection Outcomes and Their Diagnoses

Table 1 recapitulates the currently accepted PCVDs together with their major clinical signs and individual diagnostic criteria. Overall, clinical descriptions of PCVDs indicated in the table have not changed over the years and are described elsewhere [1].

PCV-2-SD is still considered as an emerging disease by most textbooks and review articles, but the very first description as postweaning multisystemic wasting syndrome (PMWS) in 1991 [14,20] took place more than 30 years ago. Moreover, evidence of PCV-2 infection and PCV-2-SD diagnosis in swine have been retrospectively demonstrated since 1962 and 1985, respectively [21]. Therefore, there is no need to consider porcine circovirus diseases (PCVDs) as emerging anymore. PCVDs must be viewed as endemic diseases with different impacts depending on the PCV-2 epidemiological and immunological herd status. In addition, PCV-2-SD is occasionally diagnosed in vaccinated pigs, this fact should remind the multifactorial causality of the disease [22] and the need to counteract such triggering factors in the farm.

The discovery of PCV-2-SI in postweaning animals was associated with the use of piglet vaccination in farms without overt clinical disease [2]. This effect prompted the widespread use of vaccines all over the world, independent of the clinical status. At that time, it was also the reason for which PCV-2 infection diagnosis was considered to not be needed anymore as part of the decision-making process to decide vaccination or not. Such rationale paralleled with the extremely great results on counteracting PCV-2-SD by means of vaccination [6,7,8].

The frequency of PCV-2-RD presentation has been traditionally considered low or very low, mainly due to the existing herd immunity at sow level [23]. However, the number of intrauterine infections can be very variable (depending on the farm) when measured through PCR methods. In addition, evidence of fetal or newborn infection can be rather high in some cases despite the lack of reproductive clinical signs [24]. While the outcome of PCV-2-SI in postweaning pigs is well-established and measurable, no solid data exist regarding subclinical infection on the reproductive side. Some studies tried to determine the impact of such intrauterine infections in farms with no overt reproductive clinical signs, but unequivocal, conclusive and reproducible effects have not been sufficiently documented [25,26]. Therefore, the impact of the subclinical PCV-2 intrauterine infections is yet to be elucidated.

Porcine dermatitis and nephropathy syndrome (PDNS) is still considered a PCVD. However, after 30 years since its first description [13] and 20 years of its proposed association with PCV-2 [27], no definitive etiological causality has been demonstrated. Evidence of PCV-2 association with PDNS is strong but circumstantial [28]. The worldwide sudden decrease in case descriptions coinciding with massive PCV-2 vaccination is probably the strongest link of PDNS with this viral infection. Although claimed to be reproduced experimentally by means of infection by *Torque teno sus virus 1a* (TTSuV1a) plus *Porcine reproductive and respiratory syndrome virus* (PRRSV) [29] and *Porcine circovirus 3* (PCV-3) [30], none of these descriptions fitted grossly and microscopically with original reports of PDNS.

Be aware that some special conditions linked to PCV-2 have been proposed in the last 20 years, such as PCV-2-lung disease (PCV-2-LD), PCV-2-enteric disease (PCV-2-ED) and acute pulmonary edema (APE) (reviewed in Segalés [1]). These are not considered in the present review. PCV-2-LD and PCV-2-ED were subsequently proposed as part of PCV-2-SD because they did not offer significant deviations from the systemic infection diagnostic criteria [31,32] and APE lasted as a single, non-further described condition [33].

## 4. Diagnostic Tools for PCV-2 Infections

### 4.1. Diagnostic Criteria for PCVDs

A PCVD diagnosis does not rule out the participation of other viral, bacterial, parasitic or fungal agents worsening the clinical outcome [34]. In consequence, the wisest recommendation in front of a suspicion of PCVD is trying to establish an integral, global diagnosis, evaluating the different etiological and risk factors involved in the clinical case. It is worth reminding that the most severe cases of PCVD documented in the literature include concomitant infections with several pathogens, with special emphasis on PRRSV [35,36].

Based on the multifactorial causation of PCVDs indicated above, it is paramount to establish solid, precise and unequivocal criteria to confirm the diagnoses of these conditions, plus assuming that the clinical outcome might not be exclusively attributed to PCV-2. These criteria have been widely presented already and extensively summarized in Segalés [1]. All in all, three steps must be considered: (1) clinical and gross pathological appearance, in the case of PCV-2-SD also being associated with a proper epidemiological presentation fitting with the so-called “herd case definition” [37]; (2) presence of specific moderate to severe histological lesions in target tissues of affected pigs, being lymphoid tissues for PCV-2-SD, heart for PCV-2-RD and vessels/glomeruli for PDNS; and (3) presence of a moderate to high amount of PCV-2 in these target tissues. The third criterion is not currently applicable to PDNS, in which PCV-2 amount tend to be low when detectable. Detection of PCV-2 in tissues is usually accomplished with techniques that allow correlating the presence of histological lesions and the presence of virus, such as immunohistochemistry (IHC) or in situ hybridization (ISH) [10].

Detection of lesions with moderate to high severity and a moderate to high amount of viral antigen (IHC) or genome (ISH) is not devoid of subjective interpretation. Moreover, practical histopathological diagnosis work over the years have shown us “unexpected” combinations in which apparently severe lesions are accompanied by a scarce amount of PCV-2 or mild lymphoid lesions harbor a high amount of the virus. However, globally speaking, those are exceptions rather than rules. There is no sound explanation for these rare cases, but they might be due to the specific combination of timing of infection, immune status of the animal, genetic background or other unknown individual factors.

In summary, the abovementioned diagnostic criteria would still be in force, especially if the diagnostic approach includes: (1) right samples for each PCVD; (2) the right clinical moment of animal selection (acute-to-subacute evolution of affected animals, not chronically affected ones); and (3) the use of several animals instead of one single animal or sample for the diagnostic attempt.

### 4.2. Diagnostic Usefulness of PCR Methodologies

One of the most widely used techniques to detect PCV-2 genome is the PCR, and especially the real-time quantitative PCR (qPCR). The advantage of qPCR is the fact that it allows the establishment of the absolute quantification of viral nucleic acid and as indicated above, the amount of PCV-2 in tissues is key for differentiating clinical PCVDs from PCV-2-SI [1]. This fact is even more important considering the widespread nature of the virus and the possibility to detect it in many samples from infected pigs. PCV-2 nucleic acid has been detected in blood, tissues, colostrum, semen, saliva as well as nasal, fecal and urinary secretions [22,38,39,40] but also from the environment (air, manure, water, sow and piglet axillary skin surfaces, gestation crate floor and bar surfaces) [41,42,43] and other animal species present in pig farms (rodents, invertebrates) [44,45]. Altogether, a first issue that should be discussed is the convenience of using a qPCR for diagnosing PCVDs and/or for strict monitoring purposes.

Disease diagnosis implies going beyond a simple detection of a pathogen, and this is very much applicable if such an agent is ubiquitous. Therefore, the use of qPCR to help diagnosing PCVD must imply to couple it with unequivocal facts characterizing these conditions. Therefore, the coincidence of a compatible clinical picture with the corresponding histopathological lesions (see Table 1) definitively points out to a potential PCVD. In such a scenario, the use of qPCR and the detection of moderate to high PCV-2 loads in systemic locations (serum or tissues) would also be confirmatory of PCVDs, as it happens with IHC or ISH. A subsequent key question is how much are moderate to high viral loads by means of qPCR.

Different studies have proposed qPCR thresholds in serum as indicative of PCV-2-SD diagnosis. These values varied among studies, including 10^4.7^ [46], 10^6.91^ [47], 10^7^ [15,17,22] and 10^6.21–7.43^ [48] viral copies/mL serum. Moreover, one study proposed qPCR thresholds in lymphoid and non-lymphoid tissues (10^6.8–8.4^ viral copies/g tissue) to diagnose PCV-2-SD [46]. In addition, a significant variation in qPCR detection limits and sensitivity has been demonstrated among laboratories [49,50]. Therefore, the abovementioned thresholds are specific of these particular qPCR methods and are not able to be generalized to whatever qPCR technique. Importantly, most of the currently used qPCR techniques correspond to commercial ones that usually offer a higher sensitivity that the ones indicated in the abovementioned papers (unpublished results). In consequence, it is very likely that the threshold to potentially discriminate clinical versus subclinical PCVDs should be at least 10^8^ PCV-2 copies/mL of serum or probably higher. Nevertheless, in case of using qPCR as a diagnostic approach, it must be coupled with clinical signs and histopathological data. If not, the sole qPCR approach should be considered as a monitoring tool rather than a diagnostic one.

Diagnostic tips for other PCVDs by means of qPCR are much more limited compared to PCV-2-SD.

PCV-2-SI is diagnosed when no overt clinical signs are present in the farm despite the virus is circulating and minimal or no histopathological lymphoid lesions are found in examined pigs [1]. This scenario has been described by many authors and qPCR results have been usually variable but mostly below the previously mentioned threshold values. However, it is very likely that, at the individual level, some non-overtly diseased animals may have viral loads above the threshold, whereas fully PCV-2-SD diagnosed by classical criteria may display values below the threshold. This was already described in the very early descriptions of qPCR methods [15,17]. Considering the ubiquitous distribution of PCV-2 among swine farms worldwide, one must conclude that the diagnosis of PCV-2-SI *per se* is poorly informative, and the use of qPCR in farms with no overt clinical signs should be focused on viral monitoring purposes. A very similar conclusion could be drawn regarding the usefulness of qPCR and PDNS, since the viral load found in these animals is comparable to the PCV-2-SI [15,51].

The diagnosis of PCV-2-RD with the aid of qPCR should probably follow a similar approach to that of PCV-2-SD, although it is likely more complicated. An early study proposed a high viral load (>10^7^ PCV-2 genome copies/500 ng DNA) in tissue samples (myocardium, spleen, and liver) of mummified or stillborn piglets and a moderate load (>10^5^ PCV-2 genome copies/500 ng DNA) in newborn piglets with myocarditis to diagnose PCV-2-RD [12]. Interestingly, only those values with ≥10^10^ genome copies/500 ng DNA in myocarditis cases yielded positive results by IHC, while those with ≤10^5^ genome copies/500 ng extracted DNA were negative for viral antigen. Subsequently, another work obtained very similar results, and values with ≥10^8^ genome copies/500 ng DNA correlated with positive IHC results. However, a significant number of fetuses with non-damaged hearts also had positive qPCR values and negative IHC results. In the latter case, the authors concluded that PCV-2 IHC positivity in fetal hearts was present in acute stages of reproductive failure, whereas qPCR was found as a more sensitive diagnostic method within a wider time span [18]. A third study has been recently published suggesting that a threshold of 10^9^ genome copies/g heart [16], coinciding with positivity by ISH, would be the most adequate threshold to fulfil the moderate to high viral load criteria indicated in Table 1. Overall, these results should not be surprising since IHC and ISH correlate with the presence of lesions and, therefore, clinical disease, while qPCR can detect both clinical and subclinical PCV-2 infections. Therefore, the specific qPCR threshold to differentiate PCV-2-RD from subclinical reproductive infections is, again, depending on the qPCR method used (as previously mentioned for PCV-2-SD), and it fits well with 10^7^ genome copies/500 ng DNA when using the oldest published techniques and with 10^9^ genome copies/g heart tissue with the one indicated in Unterweger et al. [16]. Note that some studies use ng of extracted DNA versus g of tissue by the third one, but comparison using these different methods has not been performed yet.

The use of qPCR has been expanded to other sample types such as oral fluids, placental umbilical cords, processing fluids or tissues and environmental samples [26,43,52,53]. This approach is excellent for monitoring, surveillance and epidemiological purposes, but it does not allow the establishment of disease diagnoses.

### 4.3. Diagnostic Usefulness of Antibody Detection Methodologies

PCV-2 antibodies in pigs can be generated by natural infection, transfer of maternally derived antibodies from the sow to the piglet or vaccination. Moreover, since PCV-2 vaccination is widely spread all over the world [2], the presence of antibody titers/values in fattening or adult pigs is likely to be the combination of both vaccination and natural infection. Since the origin of these antibodies cannot be traced back (lack of vaccine products that allow the differentiation of antibodies generated by infection or vaccination), the usefulness of antibody detection is limited.

The detection of PCV-2 antibodies by means of serological methodologies (ELISA tests, immunoperoxidase monolayer assay, immunofluorescence assay or others) does not provide insights in the diagnosis of PCVDs. However, these techniques, independent of the matrix used for analyses (serum, oral fluid, or meat juice) are extremely valuable for monitoring and surveillance [40,48,53,54].

## 5. PCV-2 Epidemiology and Vaccination

### 5.1. Changing PCV-2 Epidemiology

The epidemiology of PCV-2 has been modified substantially since the advent of vaccines and their massive use worldwide [2]. Despite the fact that the virus is still ubiquitous in herds, the infection pattern, viral loads, percentage of infected animals with natural infection and disease presentation have changed to the point that, eventually, one may generate batches of animals with minimal or no exposure to the virus from weaning to slaughterhouse [9]. In fact, in a short-term attempt of “PCV-2 eradication”, the virus was not able to be detected by PCR and animals did not seroconvert after six months of massive PCV-2 vaccination in sows and piglets [55].

The consequences of such epidemiological change have been seldom studied, and one might assume that the use of the piglet only, piglet and gilt, gilt/sow only or pig plus gilt/sow vaccination should yield different outcomes regarding PCV-2 infection in the mid–long term. For example, when only piglet vaccination is used, there is an increased likelihood of generating seronegative pigs at the end of the fattening period [9]. A proportion of these animals may eventually become replacement stock, therefore generating a pool of seronegative breeding stock, potentially susceptible to the viral infection. This situation fits well with the significant variability in serological values of gilts and sows that can be found in herds that vaccinate piglets only [56]. Hence, with time, continuous piglet vaccination likely generates immunologically diverse “subpopulations” of gilts/sows at the herd level. This occurs despite the fact that sows were vaccinated against PCV-2 when they were piglets at the age of 3–4 weeks, since the duration of immunity of such vaccines are considered to last around 6 months [2].

Considering that PCV-2 is present in almost all pig farms worldwide, the possibility of those “naïve” sows getting an infection is highly plausible. Although the overall rate of sow infection is usually low [56], it is expected that such a rate would be higher in animals devoid of immunity, as it has been demonstrated by the descriptions of PCV-2-RD basically in start-up herds [11,57]. Following this rationale, having susceptible gilts and sows in the farm would parallel with evidence of intrauterine infections in a proportion of breeding stock individuals. Consequently, this situation would cause the delivery of already infected newborn piglets, as has been extensively documented both under natural and experimental conditions [24,58,59]. Therefore, the herd may experience a certain proportion of early infections before PCV-2 vaccination of piglets takes place.

Overall, the proportion of infected gestating sows and gilts as well as newborns would vary between farms, and to date there is no way to predict such percentage based on the herd immunity level. Moreover, the impact of such infections on the overall performance of the farm and on the individually infected sows/gilts and piglets is unknown.

### 5.2. PCVDs in Vaccinated Herds

PCV-2 vaccines are considered very effective products, probably some of the best ones used in pigs, which would explain their ability to prevent PCV-2-SD very efficiently and their widespread use over the world [2]. However, occasional cases of PCV-2-SD have been described in PCV-2-vaccinated herds during last decade [60,61]. Actually, there is a perceived increase in these cases in the last few years and with potential association with PCV-2d, since it is the most dominating genotype lately [62]. Again, it is important to remember that PCVDs are multifactorial diseases; thus, the fact that overt disease is occurring in a herd is due to the presence of the PCV-2 infection plus the concurrence of disease triggering factors [28]. It could also happen that vaccination is not working properly in a farm, but the lack of triggering elements does not prompt the observation of overt but subclinical disease. In these cases, continuous vaccination of animals might not provide the efficiency expected for PCV-2 vaccines and remains unnoticed.

There are several reasons by which a PCV-2-vaccinated herd experiences PCV-2-SD despite vaccination, including (1) lack of real vaccination, (2) improper vaccine application, (3) late vaccination (natural infection takes place before or during vaccine application), (4) early vaccination (potential interference of maternally derived immunity or lack of a properly matured piglet immune system), and (5) vaccination in face of concomitant infection by immunomodulatory pathogens.

The first two possibilities are linked with inadequate management of the vaccination or simply that a given group of animals have not really been vaccinated. Although both situations should happen accidentally, it is important to rule them out in those cases of putative vaccination failure.

**Figure 1:**
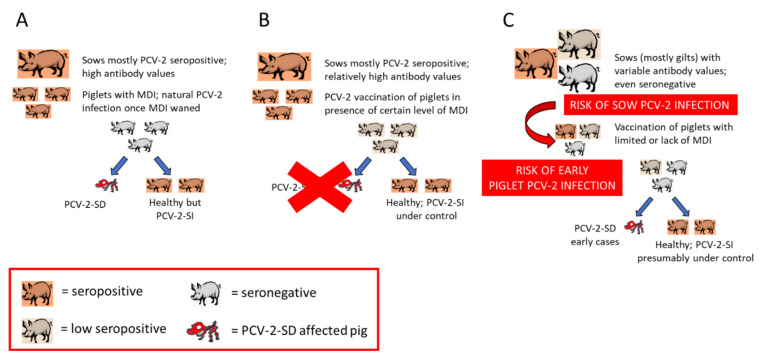
Effects of PCV-2 vaccination on the epidemiology of this viral infection. (**A**) Pre-vaccination scenario: farms infected with PCV-2 with corresponding triggering factors experienced PCV-2-SD in a proportion of pigs. (**B**) Initial vaccination scenario: PCV-2-SD is efficiently controlled by means of systematic vaccination of piglets. (**C**) Continuous vaccination scenario: Slow generation of subpopulations of naïve gilts/sows, promoting sows and offspring with relatively low immunity levels due to the lack of challenge with PCV-2. Altogether this would increase the risk of intrauterine and early piglet infections and eventual PCV-2-SD despite vaccination.

Late PCV-2 vaccination would mean that the vaccine is applied once the natural viral infection is already established. Therefore, there is no sufficient time for the vaccine to elicit proper immune responses before the virus induces disease (within the abovementioned multifactorial PCV-2-SD triggering scenario). Vaccination of relatively old animals (beyond 5 weeks of age) is very unlikely as common practice implies vaccine application around weaning (3–4 weeks of age). In fact, the scenario of late vaccination (in terms of infection prior to immunization) seems to be linked to the previously mentioned epidemiological change (Section 5.1, Figure 1). The sequence of events would be as follows. Previously to the advent of PCV-2 vaccines, those farms with infection and concurrence of triggering factors experienced PCV-2-SD in a proportion of pigs (Figure 1A). When vaccines against PCV-2 started to be applied in piglets of a diseased farm, PCV-2-SD was efficiently controlled (Figure 1B). The continuous vaccination over time would offer efficient results but would also slowly generate subpopulations of naïve gilts/sows as indicated above, promoting sows and offspring with relatively low immunity levels. This would imply an overall decrease in infectious pressure in the mid–long term, lower herd immunity levels and subsequent increased risk of intrauterine and early piglet infections. The concurrence of disease-triggering factors together with early infection in pigs before or at the time of vaccination would result in cases of PCV-2-SD (Figure 1C). The overall result is to experience the systemic disease in already vaccinated piglets. Noteworthy, those diseased cases usually appear between 6–8 weeks of age, relatively earlier than those happening before the advent of vaccines [48].

PCV-2 piglet vaccination is performed in face of certain levels of antibodies against the virus. Therefore, piglet maternally derived immunity (MDI) is overcome by the immune response generated by the vaccines, favoring the generation of piglets’ own immunity. Even though levels of MDI of the piglet at vaccination time are not homogeneous within a batch or among batches, immunization around weaning is a general profitable schedule. Although the higher the antibody levels at the time of vaccination, the lower seroconversion elicited by vaccines [63,64], the potential detrimental effect of such values on pig performance seems to be low or absent [65]. However, a study found that an apparent interference of vaccine efficacy (VE) on average daily weight gain (ADWG) was noticed in a small subpopulation of pigs with very high antibody ELISA S/P values [66]. Since the proportion of affected animals was rather low, the likely impact of the potential interference of MDI on VE under field conditions is probably negligible for most farms but not necessary for all. The degree of the potential interference effect would theoretical be higher if the piglets are vaccinated at earlier times than usual. In that sense, Haake et al. [67] found a significant difference in efficacy when comparing piglet vaccination at 1 or 3 weeks of age. The authors claimed that other age-related factors affecting the active and passive transfer of immunity instead of antibody levels would have accounted for the lack of VE in the 1-week-old vaccinated group. However, the PCV-2 antibody titers observed at three weeks of age in this study were definitively lower than those present at one week of age [67]. In consequence, the MDI interference must also be considered as a putative cause of lower vaccine efficacy in very young animals.

Pigs can be infected by several pathogens able to modulate the immune responses [68]. Data regarding the potential effects of these infectious agents or even other non-infectious factors regarding PCV-2 VE are scarce. However, one study showed that PRRSV infection at the time of PCV-2 vaccination significantly jeopardized the cellular, but not the humoral, response elicited by the immunization [69]. Consequently, it is expectable that PRRSV viremia in piglets at PCV-2 vaccination would result in an incomplete protection and underperforming VE. Interestingly, this effect was not observed in an experimental study [70]. If this effect depends on the PRRSV strain, it is yet to be elucidated.

Since PCV-2 vaccination in sows is much less frequently performed, no information exists about scenarios in which sow immunization might be detrimentally affected for any infectious or non-infectious factors. In any case, the removal of vaccination in a farm experiencing PCV-2-RD resulted in the reappearance of the reproductive disorders [19].

## 6. Discussion

Diagnostic criteria to diagnose PCV-2-SD have not changed since initial proposals [71,72], which were further compiled and revised subsequently [1]. Therefore, after 10 years of this latter revision, it is worthy to revise and update existing knowledge on PCVD diagnostic approaches, especially in the face of a massive PCV-2 vaccination scenario all over the world and the epidemiological changes this viral infection has experienced.

Three major outcomes of PCV-2 infection must be considered in practical terms: PCV-2-SI, PCV-2-SD and PCV-2-RD. While the subclinical form has always been the dominating outcome, it was not noticed until vaccines reached the market. This means that when severe outbreaks of PCV-2-SD occurred worldwide [73], the proportion of subclinically infected pigs was virtually all the remaining animals not displaying the systemic disease (i.e., 20–30% prevalence of PCV-2-SD in a batch would imply 70–80% of PCV-2-SI pigs). Since vaccines can control the subclinical effects of the infection, no real efforts have been focused on the diagnosis of this condition. However, despite the fact that PCV-2 vaccines are very efficient, they are not perfect, and they can prevent clinical disease but no infection [74]. Therefore, a proportion of vaccinated pigs still become infected during the production period although such infections seem to not impact on the ADWG [75]. However, whether a particular PCV-2 load threshold in serum is correlated with detrimental effects in pigs with no overt clinical signs at the individual level, is not known. Considering that the genetic background of the pig matters for PCV-2-SD expression [76,77,78] and viral load in serum [79], it is very likely that a given viral load might not have the same consequences on performance depending on individuals, genetic lines or breeds.

The diagnostic criteria for the systemic and reproductive diseases are well-established and initial descriptions (reviewed in Segalés et al. [22]) are still considered gold standard. In both cases, the triad composed by clinical signs, lesions and viral detection in lesions allows the diagnosis of these conditions, even animals can be concomitantly affected with other diseases. It is important to note that the presence of fetal myocardial lesions in PCV-2-RD can be variable despite finding moderate to high mounts of PCV-2 by ISH or qPCR [16,17,18,19]. This situation poses reasonable doubts on the value of histological lesions to diagnose PCV-2-RD, but so far myocarditis is the most consistent lesion observed in this condition. Therefore, the authors of the present review consider that myocardial damage should still be included within the case definition although not as a compulsory element, but as a strong support allowing the confirmation of the disease.

These individual case definitions have not been modified despite the fact that PCVDs are well-controlled nowadays by means of vaccination. What has changed over the years, and as product of technological advances, is the use and interpretation of qPCR results as a proxy for diagnosing these diseases. While early reports using mostly in-house developed molecular biology techniques proposed cut-off thresholds of 10^7^ PCV-2 genome copies per mL of sera and 500 ng of extracted DNA to diagnose PCV-2-SD and PCV-2-RD, respectively [1], current commercial qPCR methodologies have not been properly validated in that sense. Only recently, one work proposed 10^9^ viral genome copies per g of heart tissue to help diagnose PCV-2-RD [16]. The comparison of older qPCR methodologies with the currently used ones to set a new threshold for PCV-2-SD has not been performed to date, but it would be expected that a higher threshold is advisable. It is worth reminding that the viral load in target tissues, such as lymphoid organs (but also lungs), is always higher than in the serum of an infected animal. The potential threshold to suspect PCV-2-SD has been established in serum, but not in tissues; therefore, qPCR results in tissues must be taken cautiously since the 10^7^ PCV-2 genome copies per mL probably corresponds to a PCV-2-SI. In addition, the detection of viral genomes does not mean that the virus is infectious. However, if high amounts of PCV-2 nucleic acid are detected by qPCR in the tissues of a sick animal with characteristic histological lesions in lymphoid tissues, these data correlate well with the detection of viral antigens or genomes detected by IHC and ISH [15,16,17].

No further developments have occurred in relation to PDNS diagnosis. Circumstantial evidence links this condition to PCV-2 [28], but no unequivocal causality has been established yet. Therefore, PDNS diagnosis is still considered an immune-complex disease diagnosed based on gross and microscopic lesions [1].

Although qPCR and antibody detection methods are widely used to monitor PCV-2 infection, and vaccination is used massively, there are still several situations in which a complete set of diagnostic criteria for PCVD should be required. First, when the farm is experiencing overt disease compatible with PCVDs (i.e., wasting in the postweaning area or late-term reproductive problems). Second, when there are general problems of poor-doing pigs or unspecific reproductive disease, and the idea is to rule out PCVDs. Third, and the most important nowadays, when there are reasonable doubts that the vaccine is underperforming and a clear-cut diagnosis of PCVD is needed. In this latter scenario, the full confirmation of the diagnosis must be the first step to consider changing the PCV-2 vaccination scheme and to deepen the monitoring surveillance of viral infection dynamics on the farm. Major emphasis should be placed on intrauterine and/or early life infections as well as antibody value variability in the sow herd.

## 7. Conclusions

The present review aimed to update diagnostic criteria (and tools) for PCVDs, their practical implications, usefulness, limitations and interpretation. Since PCVDs are multifactorial conditions, the mere detection and even quantification of the virus is not enough to establish solid evidence of PCV-2 causality of a given clinical problem. However, to tackle the epidemiological context of a viral disease, establishment of the diagnosis is a first step, but properly monitoring the infection is as important as such diagnosis.

## Figures and Tables

**Table 1:** Summary of major clinical signs of porcine circovirus diseases (PCVDs) together with their individual case definition based on clinical and laboratorial findings (adapted from Segalés [1]).

PCVD (Acronym)	Major Clinical Signs	Individual Diagnostic Criteria
PCV-2 subclinical infection (PCV-2-SI)	Decreased average daily gain (approx. 10–40 g/day) without any evident clinical sign	Lack of overt clinical signs No or minimal histopathological lesions in tissues (mainly lymphoid) Low amount of PCV-2 in few (lymphoid) tissues, usually in follicular areas Criteria 2 and 3 can potentially be substituted by PCV-2 detection techniques such as standard PCR
PCV-2 systemic disease (PCV-2-SD)	Wasting, weight loss, decreased rate of weight gain clinically evident, ill thrift or poor-doing animals, sometimes with respiratory and/or digestive disorders	Weight loss and paleness of skin (respiratory and/or digestive clinical signs may be present as well) Moderate to severe lymphocyte depletion with granulomatous inflammation of lymphoid tissues (plus granulomatous inflammation in other tissues) Moderate to high amount of PCV-2 in lymphoid tissues (the amount in the rest of affected tissues can be variable)
PCV2 reproductive disease (PCV-2-RD)	Abortions or mummifications	Reproductive failure at late gestation or SMEDI-like condition * Fibrous to necrotizing myocarditis of fetuses Moderate to high amount of PCV-2 in the heart
	Regular return-to-estrus	Regular return-to-estrus/infertility PCV-2 seroconversion following the return-to-estrus and/or PCV-2 PCR/qPCR positivity around return-to-estrus occurrence
Porcine dermatitis and nephropathy syndrome (PDNS) **	Dark red papules and macules on skin, mainly in hind limbs and perineal area	Hemorrhagic and necrotizing skin lesions and/or swollen and pale kidneys with generalized cortical petechia Systemic necrotizing vasculitis, and necrotizing and fibrinous glomerulonephritis

* SMEDI stands for stillbirth, mummification, embryonic death and infertility; infertility would apply to return-to-estrus scenarios. ** PCV-2 association with PDNS is still circumstantial, and detection of the virus is not considered into its diagnostic case definition.

## Data Availability

Not applicable.
